# Disparities in breast cancer mortality among Latin American women: trends and predictions for 2030

**DOI:** 10.1186/s12889-023-16328-w

**Published:** 2023-07-28

**Authors:** J. Smith Torres-Román, Jorge Ybaseta-Medina, Silvana Loli-Guevara, Janina Bazalar-Palacios, Bryan Valcarcel, Miguel A. Arce-Huamani, Christian S Alvarez, Yamilee Hurtado-Roca

**Affiliations:** 1 Cancer Research Networking, Universidad Científica del Sur, Lima, Peru; 2 Latin American Network for Cancer Research (LAN–CANCER), Lima, Peru; 3 Universidad Nacional San Luis Gonzaga de Ica, Ica, Peru; 4 Sociedad Científica San Fernando, Universidad Nacional Mayor de San Marcos, Lima, Peru; 5 Universidad Tecnológica del Peru, Lima, Peru

**Keywords:** Breast cancer, Mortality, Latin America, Caribbean Region, Forecasting

## Abstract

**Background:**

Breast cancer is among the leading cause of cancer-related mortality among Latin American and Caribbean (LAC) women, but a comprehensive and updated analysis of mortality trends is lacking. The objective of this study was to determine the breast cancer mortality rates between 1997 and 2017 for LAC countries and predict mortality until 2030.

**Methods:**

We retrieved breast cancer deaths across 17 LAC countries from the World Health Organization mortality database. Age-standardized mortality rates per 100,000 women-years were estimated. Mortality trends were evaluated with Joinpoint regression analyses by country and age group (all ages, < 50 years, and ≥ 50 years). By 2030, we predict number of deaths, mortality rates, changes in population structure and size, and the risk of death from breast cancer.

**Results:**

Argentina, Uruguay, and Venezuela reported the highest mortality rates throughout the study period. Guatemala, El Salvador, and Nicaragua reported the largest increases (from 2.4 to 2.8% annually), whereas Argentina, Chile, and Uruguay reported downward trends (from − 1.0 to − 1.6% annually). In women < 50y, six countries presented downward trends and five countries showed increasing trends. In women ≥ 50y, three countries had decreased trends and ten showed increased trends. In 2030, increases in mortality are expected in the LAC region, mainly in Guatemala (+ 63.0%), Nicaragua (+ 47.3), El Salvador (+ 46.2%), Ecuador (+ 38.5%) and Venezuela (+ 29.9%).

**Conclusion:**

Our findings suggest considerable differences in breast cancer mortality across LAC countries by age group. To achieve the 2030 sustainable developmental goals, LAC countries should implement public health strategies to reduce mortality by breast cancer.

**Supplementary Information:**

The online version contains supplementary material available at 10.1186/s12889-023-16328-w.

## Introduction

In 2020, breast cancer was one of the most frequently diagnosed and deadliest malignancies in women worldwide, with more than 2 million new cases (24.5% of all cancers) and more than 600,000 deaths (15.5% of all cancers) [1], mainly affecting middle- and low-income countries [2].

Breast cancer is also the most frequent neoplasm among women in Latin America and the Caribbean (LAC) (except in Bolivia)[1, 3], with an increase in both the incidence and mortality rates [1, 4]. Since the 1990s, there has been a steady increase in breast cancer mortality rates worldwide, with the highest rates being found in LAC [5]. However, the increase in incidence and mortality in LAC has been heterogeneous in the different countries. For example, between 1992 and 2012, Brazil showed an increase in breast cancer mortality, mainly in the age group of 50 to 69 years [6]. While in Colombia, in 2018, breast cancer was the leading cause of death from cancer (mortality rate of 10.48 per 100,000 persons) [7]. Finally, from 2003 to 2017, the average mortality rate in Peru was 9.97 per 100,000 women [8].

These geographic variations in breast cancer mortality may be due to socioeconomic status, lifestyles, and access to timely care in each country [9–12]. Premenopausal women were found to have a higher risk of breast cancer than age-matched postmenopausal women [13–15].

Previous studies in the LAC population have been limited to short periods of time and a limited number of countries, not allowing exhaustive comparison of the evolution of breast cancer trends in this region. In addition, it is important to know how mortality rates have evolved in the last two decades and what patterns will follow in the coming years, with the goal of proposing containment measures for each country in order to reduce mortality by this neoplasm. Therefore, our objective was to provide breast cancer mortality rates between 1997 and 2017 for LAC countries and predict their mortality rates for the year 2030.

## Materials and methods

### Data source

We conducted an observational study with an ecological design. The World Health Organization (WHO) mortality database was used, which compiles death registration data from member states and includes information on causes of death. This mortality database is freely accessible at the following link: https://www.who.int/data/data-collection-tools/who-mortality-database.

Mortality data for LAC countries were extracted for the period from 1997 (or first available year) to 2017 (or latest available year). Breast cancer deaths were identified with the code C50 (ICD-10: malignant neoplasm of the breast). The analysis was performed for 5-year age intervals (0–4, 5–9, 10–14, …, 85 and over). Projected population estimates for the study countries were obtained from World Population Prospects [16].

### Age adjusted mortality rates

We estimated age-standardized mortality rates per 100,000 female-years by the direct method using the Segi world standard population [17]. The analysis was performed for 5-year age intervals (0–4, 5–9, 10–14, …, 85 and older), also divided into two groups, < 50 years and ≥ 50 years. Age-specific mortality trends were estimated using Poisson models with the Joinpoint regression program version 4.7.0. Joinpoints given by the program were identified and the annual percentage change (APC) and corresponding 95% confidence intervals (95%CI) were estimated for each country. The APC were considered statistically significant with a p-value < 0.05. We calculated the average APC (AAPC) for countries that had 2 or more attachment points.

### Predictions of mortality rates

Predictions for the year 2030 were made using the Nordpred package in the R software, based on an age-period-cohort model. An evaluation was made between the last observed period and the last projected period. The predicted data were compared with the expected rate if the rate found for the observed period was maintained in the projected period in each of the age groups evaluated. This evaluation made it possible to identify whether the difference was due to changes in the structure and size of the population or to an increased risk of death from breast cancer, as proposed by Møller et al. using the formula [18]:

$${\rm{\Delta tot}}\,{\rm{ = }}\,{\rm{\Delta risk}}\,{\rm{ + }}\,{\rm{\Delta pop}}\,{\rm{ = }}\,\left( {{\rm{Nfff}}\,{\rm{ - }}\,{\rm{Noff}}} \right)\,{\rm{ + }}\,\left( {{\rm{Noff}}\,{\rm{ - }}\,{\rm{Nooo}}} \right)$$

Where:

Δtot: The total change between the two periods analyzed.

Δrisk: The change as a function of risk.

Δpop: Is the change as a function of changes in population size and structure.

Nooo: The number of observed cases.

Nfff: The number of predicted cases.

Noff: is the number of expected cases using the rate of the observed period to the population of the last predicted period.

This study was approved by the Institutional Research Ethics Committee of the Universidad Cientifica del Sur (CIEI-CIENTÍFICA) (449-2021-POS50).

## Results

**Fig. 1:**
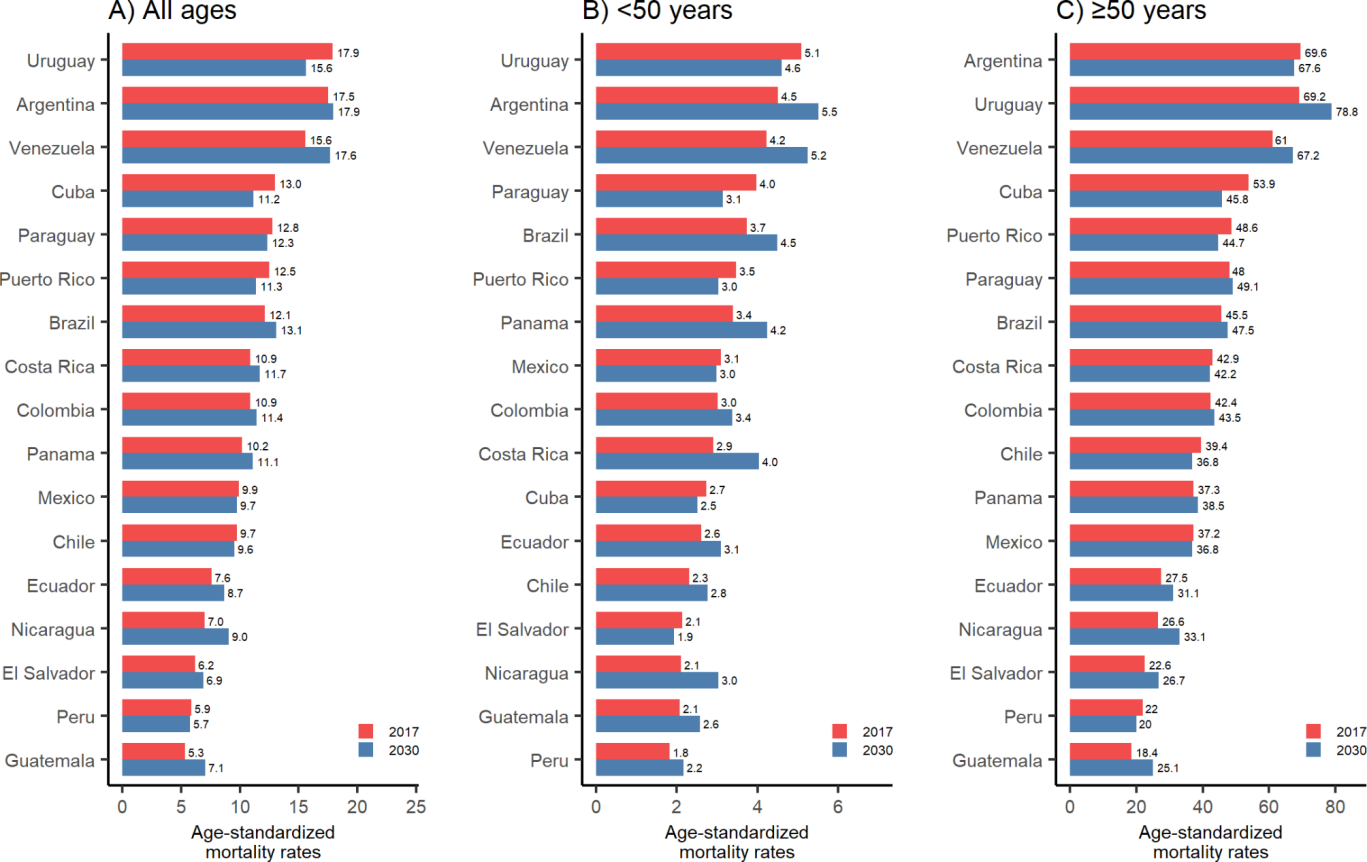
Age-standardized (SEGI’s world standard population) breast cancer mortality rates per 100,000 in 2017* and predicted for 2030** in Latin America and the Caribbean. *Data from 2016 for Venezuela. **Age-standardized rate represents the mid-point for the projected period 2028–2032

Figure 1 shows breast cancer mortality rates for the latest available year and their prediction for the year 2030 in LAC countries. In 2017, Argentina (17.5), Uruguay (17.9), and Venezuela (15.6) had the highest mortality rates for all ages, and in 2030 Argentina (17.9), Uruguay (15.6), and Venezuela (17.7) will continue to have the highest mortality rates.

**Fig. 2:**
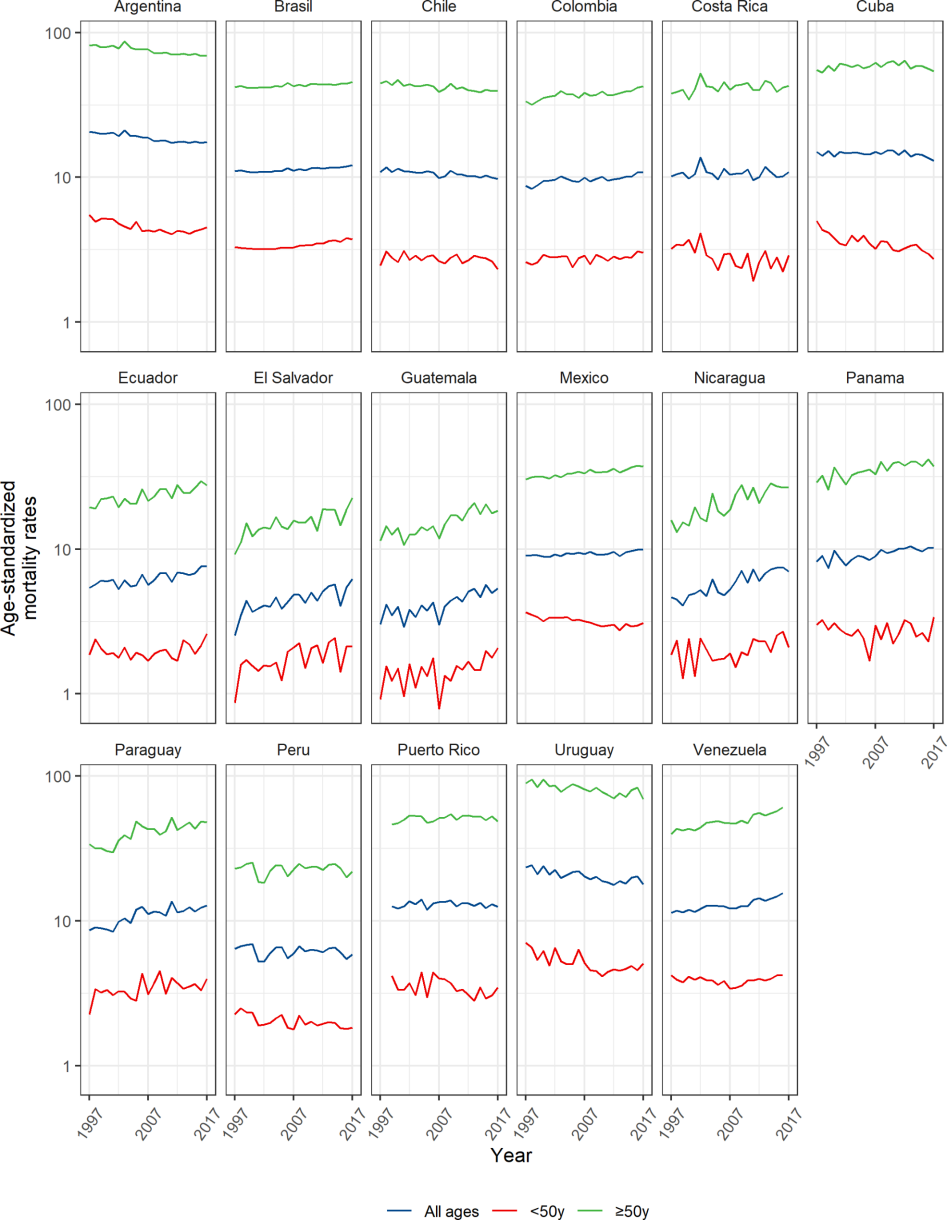
Breast cancer mortality trends (rates per 100,000 women-years, all ages, < 50 years, and ≥ 50 years) 1997–2017 in Latin America and the Caribbean

**Fig. 3:**
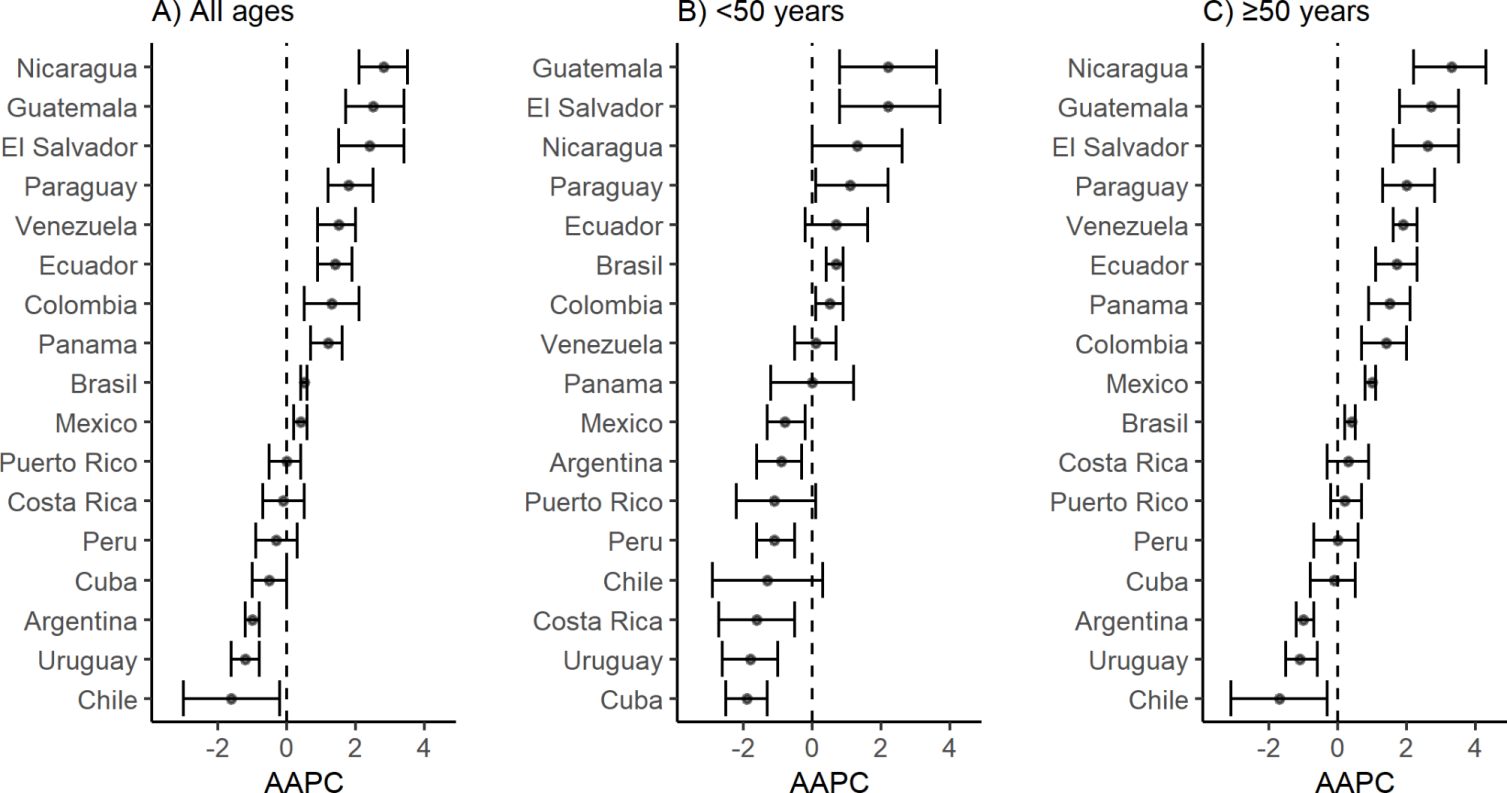
Average annual percent change (AAPC) and 95% confidence interval (CI) for breast cancer mortality rates in Latin America and the Caribbean, 1997–2017

Table 1 shows the estimated annual percentage change for age-adjusted mortality rates of breast cancer in LAC countries. Ten countries showed significant increases in their mortality rates: Brazil (+ 0.5%), Colombia (+ 1.3%), Ecuador, (+ 1.4%), El Salvador (+ 2.4), Guatemala (+ 2.5%), Mexico (+ 0.4%), Nicaragua (+ 2.8%), Panama (+ 1.2%), Paraguay (+ 1.8%), and Venezuela (+ 1.5%), while three countries reported downward trends: Argentina (− 1.0%), Chile (− 1.6%), and Uruguay (− 1.2%) (Fig. 2, **and** Fig. 3**)**.

**Table 1:** Average annual percentage change (AAPC) with their 95% confidence intervals (95%CI) for age-adjusted breast cancer mortality rates among women of all ages in Latin America and the Caribbean, 1997–2017

Countries	Calendar Years	EAPC	Period	APC	Period	APC	AAPC
Argentina	1997–2017	−1.0*(− 1.2,−0.8)					−1.0*(− 1.2,−0.8)
Brasil	1997–2017	0.5*(0.4, 0.6)					0.5*(0.4, 0.6)
Chile	1997–2017	−1.6*(− 3.0, − 0.2)					−1.6*(− 3.0, − 0.2)
Colombia	1997–2002	2.7*(0.2, 5.3)	2002–2013	0(− 0.7, 0.7)	2013–2017	3.0*(0.5, 5.5)	1.3*(0.5, 2.1)
Costa Rica	1997–2017	−0.1(− 0.7, 0.5)					−0.1(− 0.7, 0.5)
Cuba	1997–2012	0.2(− 0.2, 0.6)	2012–2017	−2.4*(− 4.2, − 0.6)			−0.5(− 1.0, 0)
Ecuador	1997–2017	1.4*(0.9, 1.9)					1.4*(0.9, 1.9)
El Salvador	1997–2017	2.4*(1.5, 3.4)					2.4*(1.5, 3.4)
Guatemala	1997–2017	2.5*(1.7, 3.4)					2.5*(1.7, 3.4)
Mexico	1997–2017	0.4*(0.2, 0.6)					0.4*(0.2, 0.6)
Nicaragua	1997–2017	2.8*(2.1, 3.5)					2.8*(2.1, 3.5)
Panama	1997–2017	1.2*(0.7, 1.6)					1.2*(0.7, 1.6)
Paraguay	1997–2017	1.8*(1.2, 2.5)					1.8*(1.2, 2.5)
Peru	1997–2017	−0.3(− 0.9, 0.3)					−0.3(− 0.9, 0.3)
Puerto Rico	1999–2017	0(− 0.5, 0.4)					0(− 0.5, 0.4)
Uruguay	1997–2017	−1.2*(− 1.6, − 0.8)					−1.2*(− 1.6, − 0.8)
Venezuela	1997–2009	0.8*(0.1, 1.4)	2009–2016	2.7*(1.6, 3.9)			1.5*(0.9, 2.0)

APC: Annual percentage change. AAPC: Average annual percentage change. * p-value < 0.05

In women under 50 years of age, the mortality rates by breast cancer in Argentina (− 0.9%), Costa Rica (− 1.6%), Cuba (− 1.9%), Mexico (− 0.8%), Peru (− 1.1%) and Uruguay (− 1.8%) significantly decreased, while Brazil (+ 0.7%), Colombia (+ 0.5%), El Salvador (+ 2.2%), Guatemala (+ 2.2%), and Paraguay (+ 1.1%) showed significant increases during the study period (Table 2; Fig. 2, **and** Fig. 3).

**Table 2:** Average annual percentage change (AAPC) with their 95% confidence intervals (95%CI) of for age-adjusted breast cancer mortality rates among women < 50 years in Latin America and the Caribbean, 1997–2017

Countries	Calendar Years	APC (CI95%)	Calendar Years	APC (CI95%)	AAPC
Argentina	1997–2017	−0.9*(− 1.6, − 0.3)			−0.9*(− 1.6, − 0.3)
Brasil	1997–2017	0.7*(0.4, 0.9)			0.7*(0.4, 0.9)
Chile	1997–2017	−1.3(-2.9, 0.3)			−1.3(-2.9, 0.3)
Colombia	1997–2017	0.5*(0.1, 0.9)			0.5*(0.1, 0.9)
Costa Rica	1997–2017	−1.6*(− 2.7, − 0.5)			−1.6*(− 2.7, − 0.5)
Cuba	1997–2017	−1.9*(− 2.5, − 1.3)			−1.9*(− 2.5, − 1.3)
Ecuador	1997–2017	0.7(− 0.2,1.6)			0.7(− 0.2,1.6)
El Salvador	1997–2017	2.2*(0.8, 3.7)			2.2*(0.8, 3.7)
Guatemala	1997–2017	2.2*(0.8, 3.6)			2.2*(0.8, 3.6)
Mexico	1997–2017	−0.8*(-1.3,-0.2)			−0.8*(-1.3,-0.2)
Nicaragua	1997–2017	1.3(-0.0,2.6)			1.3(-0.0,2.6)
Panama	1997–2017	0(-1.2,1.2)			0(-1.2,1.2)
Paraguay	1997–2017	1.1*(0.1, 2.2)			1.1*(0.1, 2.2)
Peru	1997–2017	−1.1*(− 1.6, − 0.5)			−1.1*(− 1.6, − 0.5)
Puerto Rico	1997–2017	−1.1(− 2.2, 0.1)			−1.1(− 2.2, 0.1)
Uruguay	1997–2017	−1.8*(− 2.6, − 1.0)			−1.8*(− 2.6, − 1.0)
Venezuela	1997–2008	−1.3*(− 2.1, − 0.5)	2008–2016	2.2*(1.0, 3.3)	0.1(− 0.5, 0.7)

APC: Annual percentage change. AAPC: Average annual percentage change. * p-value < 0.05

In women aged 50 years and older, mortality rates by breast cancer significantly decreased in Argentina (− 1.0%), Chile (− 1.7%), and Uruguay (− 1.1%) during the study period, while in Brazil (+ 0.4%), Colombia (+ 1.4%), Ecuador (+ 1.7%), El Salvador (+ 2.6%), Guatemala (+ 2.7%), Mexico (+ 1.0%), Nicaragua (+ 3.3%), Panama (+ 1.5%), Paraguay (+ 2.0%), and Venezuela (+ 1.9%) reported significantly increased. (Table 3; Fig. 2, **and** Fig. 3).

**Table 3:** Average annual percentage change (AAPC) with their 95% confidence intervals (95%CI) of for age-adjusted breast cancer mortality rates among women ≥ 50 years in Latin America and the Caribbean, 1997–2017

Countries	Calendar Years	APC	Period	APC	Period	APC	AAPC
Argentina	1997–2017	−1.0*(− 1.2, − 0.7)					−1.0*(− 1.2, − 0.7)
Brasil	1997–2017	0.4*(0.2, 0.5)					0.4*(0.2, 0.5)
Chile	1997–2017	−1.7*(− 3.1, − 0.3)					−1.7*(− 3.1, − 0.3)
Colombia	1997–2003	2.7*(1.0, 4.4)	2003–2012	−0.2(− 1.1,0.7)	2012–2017	2.6*(1.1, 4.1)	1.4*(0.7, 2.0)
Costa Rica	1997–2017	0.3(− 0.3, 0.9)					0.3(− 0.3, 0.9)
Cuba	1997–2012	0.7*(0.2,1.2)	2012–2017	−2.6*(− 4.9, − 0.2)			−0.1(− 0.8, 0.5)
Ecuador	1997–2017	1.7*(1.1, 2.3)					1.7*(1.1, 2.3)
El Salvador	1997–2017	2.6*(1.6, 3.5)					2.6*(1.6, 3.5)
Guatemala	1997–2017	2.7*(1.8, 3.5)					2.7*(1.8, 3.5)
México	1997–2017	1.0*(0.8,1.1)					1.0*(0.8,1.1)
Nicaragua	1997–2017	3.3*(2.2, 4.3)					3.3*(2.2, 4.3)
Panamá	1997–2017	1.5*(0.9, 2.1)					1.5*(0.9, 2.1)
Paraguay	1997–2017	2.0*(1.3, 2.8)					2.0*(1.3, 2.8)
Perú	1997–2017	0(− 0.7,0.6)					0(− 0.7,0.6)
Puerto Rico	199–2017	0.2(− 0.2, 0.7)					0.2(− 0.2, 0.7)
Uruguay	1997–2017	−1.1*(− 1.5, − 0.6)					−1.1*(− 1.5, − 0.6)
Venezuela	1997–2016	1.9*(1.6, 2.3)					1.9*(1.6, 2.3)

APC: Annual percentage change. AAPC: Average annual percentage change. * p-value < 0.05

According to the predictions for 2030, in women of all ages, most countries showed increases in mortality rates between 2017 and 2030. While decreases in breast mortality rates are predicted in 5 out of 17 countries, increases are predicted for the most Latin American and Caribbean countries, with the most substantial increases in Guatemala (+ 63.0%), Nicaragua (+ 47.3), El Salvador (+ 46.2%), Ecuador (+ 38.5%) and Venezuela (+ 29.9%) ( Table 4).

**Table 4:** Number of breast cancer deaths, age-standardized mortality rates, and percentage change in cases due to population growth and risk among women in Latin America and the Caribbean, 2017 and predicted for 2030. (all ages)

Country	Female population (million per year)		Number of deaths in women of all ages		Age-standardized mortality rates		Total change, %	Change due to population, %	Change due to risk, %
	2017	2030	2017	2030	2017	2030			
Argentina	22.60	25.07	6024	7421	17.52	17.92	27.9	26.7	1.2
Brazil	107.44	114.24	16,723	25,368	12.10	13.09	65.1	50.6	14.5
Chile	9.28	9.85	1504	1904	9.73	9.56	30.1	37.7	−7.5
Colombia	24.93	27.22	3300	5151	10.89	11.41	75.1	58.9	16.3
Costa Rica	2.45	2.74	366	593	10.90	11.67	76.9	63.6	13.3
Cuba	5.67	5.62	1519	1699	12.96	11.17	12.0	30.8	−18.8
Ecuador	8.32	9.92	667	1144	7.60	8.70	97.7	59.2	38.5
El Salvador	3.25	3.62	217	220	6.22	6.89	87.1	40.9	46.2
Guatemala	8.68	10.74	352	739	5.35	7.08	129.9	66.8	63.0
México	65.44	71.96	6755	9746	9.93	9.74	58.2	52.8	5.5
Nicaragua	3.15	3.75	202	410	7.00	9.04	112.6	65.3	47.3
Panamá	2.02	2.47	241	508	10.18	11.09	129.4	107.0	22.4
Paraguay	3.36	3.92	391	556	12.78	12.32	56.8	53.8	3.0
Perú	16.10	18.16	1030	1517	5.86	5.74	52.4	58.4	−6.0
Puerto Rico	1.91	1.53	445	492	12.51	11.35	13.0	19.4	−6.4
Uruguay	1.79	1.84	204	260	17.91	15.60	7.0	23.4	−16.4
Venezuela*	16.54	17.06	2513	1289	15.58	17.65	77.5	47.6	29.9

*Data from 2016 for Venezuela

In women < 50 years, the population structure in Cuba and Puerto Rico will decrease by the year 2030, which will positively influence the total change in mortality by breast cancer. Panama will have the greatest increase in population change and change due to risk, which will negatively influence this disease (Supplementary 1). On the other hand, in women ≥ 50 years, Cuba will show a decrease in breast cancer mortality, mainly due to a change in its population structure (Supplementary 2).

## Discussion

In this study, mortality trends for breast cancer between 1997 and 2017 were compared across LAC countries, and mortality for 2030 was predicted. Certain LAC countries recorded increases in breast cancer death rates of more than 2% annually, while others reported decreases of 1–1.6%. Between 1997 and 2027, mortality patterns from breast cancer varied significantly throughout LAC countries depending on the age cutoff of women older and younger than 50 years. The breast cancer mortality rates in Chile, Cuba, Paraguay, Peru, and Puerto Rico are expected to decrease by 2030, although these changes will not be significant.

We found that between 1997 and 2017 Argentina, Uruguay, and Venezuela had the highest breast cancer mortality rates in the region. These results reflect current trends reported by GLOBOCAN for the year 2020, which indicate that these three countries had the highest rates in the region (more than 17 deaths per 100,000) [1, 3]. Nevertheless, these rates are low in comparison to countries that have not been included in this study, such as Barbados, Jamaica and Bahamas, which report rates of more than 30 deaths per 100,000 [3]. One of the main risk factors for the development of breast cancer is age, with changes in demographic structure leading to epidemiological changes in which breast cancer will reach epidemic proportions in LAC. In addition, differences in mortality between countries could be explained by variations in prevention strategies, access to health services, health insurance coverage and patient care practices (i.e., the lack of specialists in the rural areas of each country or specialized institutes for comprehensive cancer management). The inequalities in access to breast cancer care are strikingly visible within the region, and this is evidenced by the mortality outcomes in many of the countries reported in this study.

The disparity in mortality rates between LAC countries for women over and under 50 years old remains significant. When compared to older women, younger women have worse prognoses and a higher risk of breast cancer recurrence and mortality [19, 20]. Several studies [21–23] have investigated risk variables that may affect the incidence of breast cancer in different countries. Demographic, socioeconomic, genetic, and lifestyle-related risk variables, as well as reproductive behavior, are the most important risk factors. Age is still the most important factor in the occurrence of breast cancer. Brazil, Colombia, El Salvador, Guatemala, and Paraguay showed significant increases in breast cancer mortality in women under and over 50 years old between 1997 and 2017. As most Latin American countries migrate to a more advanced population age structure, demographic shifts in the region are causing an epidemiological transition. Currently, 6.9% of the Brazilian population is 65 or older, and it is expected to double by 2030, significantly increasing the number of instances of breast cancer. Several studies have investigated the impact of behavioral and lifestyle risk factors on the increased risk of breast cancer [24], such as dietary pattern [25], physical activity [26], smoking [27], and alcohol intake [28]. On the contrary, if a very healthy lifestyle is maintained, the chance of getting breast cancer in the premenopausal and postmenopausal stages is reduced by 50% and 80%, respectively [29].The inclusion of mammography screening in primary care programs, as well as the adoption of quality standards, are likely to have played a role in improving diagnoses in these nations. Women are diagnosed earlier in Brazil, particularly in affluent regions, albeit at a far lower rate than in Sweden. Several LAC studies have found an adverse relationship between the diagnosis of late-stage breast cancer and survival [30–32]. Depending on the health organization in each country and individual health insurance, socioeconomic status might influence access to care (including access to early diagnosis and treatment with specific drugs) [33]. Access to diagnosis and treatment allows women with breast cancer to be diagnosed at an earlier stage, improving their chances of survival [34] [35].[36] More investment in health systems in LAC countries is required for early detection of breast cancer, earlier diagnosis, and accessible and inexpensive treatment for all women.

Some LAC countries are expected to see reductions in breast cancer mortality rates by 2030. Argentina, Brazil, Paraguay, Uruguay, and Venezuela will have the highest mortality rates in the area, with more than 12 deaths per 100,000 people, while El Salvador and Peru will have the lowest, with less than 6 fatalities per 100,000 people. Our estimations exceed those reported in Los Angeles and Western European countries [37]. The limited availability of screening and the time lag between initial suspicion of breast cancer and diagnosis are two characteristics that can affect clinical results, and that inferior access to treatment also has an impact on clinical outcomes [24, 38]. Latin American women are diagnosed at a younger age than women in North America and Europe [27]. Early menarche, late-term pregnancy, nulliparity, and a lack of nursing are well-known risk factors for breast cancer occurrence [39]. Furthermore, lifestyle behaviors such as nutrition and obesity, as well as environmental variables, may contribute to adverse trends in young women [39]. To improve future outcomes, programs must be structured, and new interventions welcomed.

This study presents the usual limitations of a cancer registry analysis of a secondary database, such as the quality and validity of the certification of deaths, the lack of specific information on tumor characteristics, limited information at the individual level and the lack of cases to be able to perform an incidence analysis and show a more comprehensive analysis of what is happening in relation to breast cancer in each LAC country.

## Conclusion

This is the first study to analyze breast cancer mortality rates from 1997 to 2017 and create projections beyond 2030 in LAC countries. We discovered that breast cancer death rates rose in certain countries and fell in others during the study period. Similarly, the patterns of death from breast cancer varied dramatically across LAC countries based on age. Breast cancer mortality rates are expected to fall in Chile, Cuba, Paraguay, Peru, and Puerto Rico by 2030. To achieve the 2030 sustainable developmental goals, LAC countries should implement public health strategies to reduce mortality by breast cancer.

## Electronic supplementary material

Below is the link to the electronic supplementary material.

Supplementary Material 1

Supplementary Material 2

## Data Availability

The datasets generated and/or analysed during the current study are available in the following link: https://www.who.int/data/data-collection-tools/who-mortality-database.
