# Supplementary material for: Disparities in breast cancer mortality among Latin American women: trends and predictions for 2030
Source: BMC Public Health. 2023 Jul 28;23:1449. doi: 10.1186/s12889-023-16328-w (PMC10386226; doi:10.1186/s12889-023-16328-w)
Supplement: Supplementary file 1 — Supplementary Material 1 [file 12889_2023_16328_MOESM1_ESM.doc]

Supplementary 1. Number of breast cancer deaths, age-standardized mortality rates, and percentage change in cases due to population growth and risk among women in Latin America and the Caribbean, 2015 and predicted for 2030. (<50 years)

| **Country** | **Female population (million per year)** | | **Number of deaths in women of all ages** | | **Age-standardized mortality rates** | | **Total change, %** | **Change due to population, %** | **Change due to risk, %** |
| --- | --- | --- | --- | --- | --- | --- | --- | --- | --- |
|  | **2017** | **2030** | **2017** | **2030** | **2017** | **2030** |  |  |  |
| Argentina | 16.43 | 17.40 | 838 | 1183 | 4.51 | 5.51 | 55.0 | 20.2 | 34.8 |
| Brazil | 80.31 | 76.14 | 3761 | 5026 | 3.74 | 4.49 | 39.2 | 14.3 | 24.9 |
| Chile | 6.38 | 6.18 | 197 | 249 | 2.31 | 2.76 | 10.1 | 7.0 | 3.1 |
| Colombia | 18.94 | 18.64 | 689 | 856 | 3.01 | 3.38 | 31.7 | 12.6 | 19.1 |
| Costa Rica | 1.81 | 1.83 | 63 | 109 | 2.90 | 4.04 | 89.3 | 24.5 | 64.8 |
| Cuba | 3.53 | 3.07 | 163 | 117 | 2.73 | 2.52 | −39.3 | −24.7 | −14.6 |
| Ecuador | 6.65 | 7.41 | 178 | 267 | 2.61 | 3.10 | 82.3 | 30.8 | 51.5 |
| El Salvador | 2.55 | 2.64 | 57 | 62 | 2.13 | 1.93 | 14.4 | 21.8 | −7.4 |
| Guatemala | 7.48 | 8.81 | 121 | 233 | 2.08 | 2.58 | 131.9 | 58.4 | 73.5 |
| Mexico | 52.38 | 52.10 | 1796 | 1942 | 3.09 | 2.98 | 17.9 | 16.8 | 1.1 |
| Nicaragua | 2.61 | 2.87 | 54 | 106 | 2.10 | 3.03 | 87.3 | 42.2 | 45.0 |
| Panama | 1.58 | 1.78 | 60 | 148 | 3.40 | 4.24 | 210.7 | 102.2 | 108.5 |
| Paraguay | 2.77 | 3.11 | 93 | 109 | 3.98 | 3.14 | 33.6 | 53.4 | −19.8 |
| Peru | 12.77 | 13.10 | 250 | 361 | 1.83 | 2.17 | 45.4 | 24.5 | 20.9 |
| Puerto Rico | 1.24 | 771 | 59 | 35 | 3.48 | 3.03 | −35.6 | −32.7 | −2.9 |
| Uruguay | 1.19 | 1.15 | 75 | 69 | 5.10 | 4.60 | 0.6 | 4.3 | −3.7 |
| Venezuela | 13.55 | 12.49 | 548 | 798 | 4.23 | 5.25 | 50.3 | 17.9 | 32.4 |
