# Supplementary material for: Disparities in breast cancer mortality among Latin American women: trends and predictions for 2030
Source: BMC Public Health. 2023 Jul 28;23:1449. doi: 10.1186/s12889-023-16328-w (PMC10386226; doi:10.1186/s12889-023-16328-w)
Supplement: Supplementary file 2 — Supplementary Material 2 [file 12889_2023_16328_MOESM2_ESM.doc]

Supplementary 2. Number of breast cancer deaths, age-standardized mortality rates, and percentage change in cases due to population growth and risk among women in Latin America and the Caribbean, 2015 and predicted for 2030.( ≥50 years)

| **Country** | **Female population (million per year)** | | **Number of deaths in women of all ages** | | **Age-standardized mortality rates** | | **Total change, %** | **Change due to population, %** | **Change due to risk, %** |
| --- | --- | --- | --- | --- | --- | --- | --- | --- | --- |
|  | **2017** | **2030** | **2017** | **2030** | **2017** | **2030** |  |  |  |
| Argentina | 6.17 | 7.67 | 5186 | 6237 | 69.56 | 67.57 | 23.7 | 27.6 | −3.9 |
| Brazil | 27.13 | 38.10 | 12962 | 20341 | 45.53 | 47.49 | 73.1 | 61.8 | 11.3 |
| Chile | 2.90 | 3.66 | 1307 | 1654 | 39.44 | 36.77 | 33.8 | 43.3 | −9.5 |
| Colombia | 5.99 | 8.58 | 2611 | 4295 | 42.40 | 43.51 | 87.5 | 72.0 | 15.4 |
| Costa Rica | 0.64 | 915 | 303 | 484 | 42.91 | 42.16 | 74.3 | 71.7 | 2.7 |
| Cuba | 2.14 | 2.55 | 1356 | 1581 | 53.90 | 45.79 | −39.3 | −24.7 | −14.6 |
| Ecuador | 1.67 | 2.51 | 489 | 876 | 27.52 | 31.06 | 103.0 | 68.8 | 34.1 |
| El Salvador | 0.69 | 981 | 160 | 289 | 22.60 | 26.69 | 116.8 | 48.7 | 68.1 |
| Guatemala | 1.21 | 1.92 | 231 | 506 | 18.42 | 25.08 | 128.9 | 70.7 | 58.2 |
| Mexico | 13.06 | 19.87 | 4959 | 7803 | 37.25 | 36.80 | 73.7 | 66.0 | 7.7 |
| Nicaragua | 0.55 | 875 | 148 | 304 | 26.57 | 33.10 | 123.1 | 74.8 | 48.3 |
| Panama | 0.45 | 686 | 181 | 359 | 37.31 | 38.47 | 107.0 | 108.3 | −1.4 |
| Paraguay | 0.59 | 818 | 298 | 446 | 48.00 | 49.10 | 30.7 | 57.9 | −27.2 |
| Peru | 3.33 | 5.07 | 780 | 1156 | 21.95 | 20.05 | 54.7 | 69.7 | −15.0 |
| Puerto Rico | 0.67 | 754 | 386 | 458 | 48.64 | 44.66 | 19.8 | 26.7 | −6.9 |
| Uruguay | 0.60 | 684 | 536 | 699 | 69.16 | 78.76 | 22.7 | 17.4 | 5.3 |
| Venezuela | 2.99 | 4.58 | 2186 | 3322 | 61.01 | 67.20 | 85.6 | 56.4 | 29.2 |
